# Proteasome activity inhibition mediates endoplasmic reticulum stress-apoptosis in triptolide/lipopolysaccharide-induced hepatotoxicity

**DOI:** 10.1007/s10565-024-09903-3

**Published:** 2024-07-29

**Authors:** Ruohan Cheng, Yihan Jiang, Yue Zhang, Mohammed Ismail, Luyong Zhang, Zhenzhou Jiang, Qinwei Yu

**Affiliations:** 1 Jiangsu Center for Pharmacodynamics Research and Evaluation, State Key Laboratory of Natural Medicines, New Drug Screening Center, China Pharmaceutical University, Nanjing, 210009 China; 2 Center for Drug Research and Development, Guangdong Pharmaceutical University, Guangzhou, 510006 China; 3 Key Laboratory of Drug Quality Control and Pharmacovigilance, Ministry of Education, China Pharmaceutical University, Nanjing, 210009 China

**Keywords:** Triptolide, Endoplasmic reticulum stress, Proteasome activity, Reactive oxygen species, Apoptosis

## Abstract

Triptolide (TP) is a major active and toxic composition of the Chinese medicine *Tripterygium wilfordii* Hook. F. (TWHF), exhibiting various therapeutic bioactivities. Among the toxic effects, the hepatotoxicity of TP deserves serious attention. Previously, our research group proposed a new view of TP-related hepatotoxicity: hepatic hypersensitivity under lipopolysaccharide (LPS) stimulation. However, the mechanism of TP/LPS-induced hepatic hypersensitivity remains unclear. In this study, we investigated the mechanism underlying TP/LPS-induced hypersensitivity from the perspective of the inhibition of proteasome activity, activated endoplasmic reticulum stress (ERS)-related apoptosis, and the accumulation of reactive oxygen species (ROS). Our results showed that N-acetylcysteine (NAC), a common ROS inhibitor, decreased the expression of cleaved caspase-3 and cleaved PARP, which are associated with FLIP enhancement. Moreover, 4-phenylbutyric acid (4-PBA), an ERS inhibitor, was able to alleviate TP/LPS-induced hepatotoxicity by reducing ERS-related apoptosis protein expression (GRP78, p-eIF2α/eIF2α, ATF4, CHOP, cleaved caspase-3 and cleaved PARP) and ROS levels, with ATF4 being an indispensable mediator. In addition, the proteasome activity inhibitor MG-132 further aggravated ERS-related apoptosis, which indicated that the inhibition of proteasome activity also plays an important role in TP/LPS-related liver injuries. In summary, we propose that TP/LPS may upregulate the activation of ERS-associated apoptosis by inhibiting proteasome activity and enhancing ROS production through ATF4.

**Graphical Abstract:**

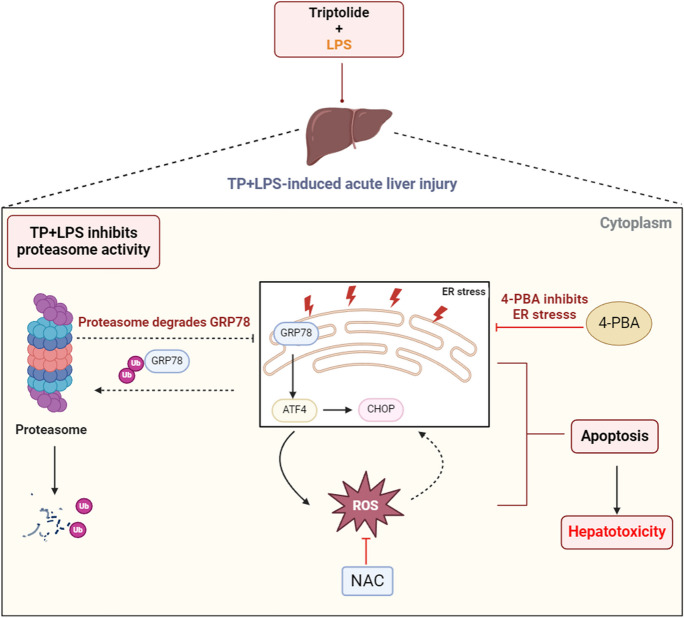

**Supplementary Information:**

The online version contains supplementary material available at 10.1007/s10565-024-09903-3.

## Introduction

Triptolide (TP) is an epoxidized diterpene lactone extraction from *Tripterygium wilfordii* Hook F. (TWHF), a traditional Chinese herb. It exhibits pharmacological effects against inflammatory, bacterial, tumor and immune systems, but it also has toxicity in the livers, kidneys, and gastrointestinal systems (Cui et al. 2023; Li et al. 2014, 2015; Qu et al. 2015). According to clinical reports, prolonged administration of TP-containing drugs can arouse severe hepatotoxicity. Nevertheless, our laboratory findings showed that consecutive 14-day administration of 600 μg/kg TP (55 times the clinical dose) to C57BL/6 mice failed to cause significant liver injury. This discrepancy suggests that direct liver damage may not be the sole underlying mechanism responsible for hepatotoxicity. Accordingly, our group has proposed a new perspective on TP-associated hepatotoxicity: TP induces increased liver sensitivity to nontoxic doses of lipopolysaccharide (LPS), which involves the inhibition of FLICE inhibitory protein (FLIP), a pro-survival protein, and the activation of apoptosis and necrotic apoptosis may be critical factors in TP/LPS-induced hepatotoxicity (Yuan et al. 2020, 2019). The above results emphasized the importance of resisting apoptosis in alleviating TP/LPS-related hepatotoxicity.

Regarding apoptosis, two most extensively researched pathways involved: one is the intrinsic apoptotic pathway via mitochondrial mediation, and the other is the extrinsic apoptotic pathway via the participation of death receptors (Green and Llambi 2015). Recently, there has been increasing interest in endoplasmic reticulum stress (ERS)-induced apoptosis, which is known as the ER-associated death pathway, involving the expression of CCAAT/enhancer-binding protein homologous protein (CHOP)/GADD153, c-Jun N-terminal kinase (JNK) and capase12 activation (Zhang et al. 2022). Reportedly, activation of the ERS-associated death pathway is linked to acetaminophen (APAP)-induced liver injury (Kusama et al. 2017). Furthermore, in rifampicin-induced cholestatic liver disease, 4-phenylbutyric acid (4-PBA) can alleviate liver injury through restraining ERS and multidrug resistance-associated protein 2 ubiquitination-mediated degradation (Chen et al. 2022). Upon encountering ERS, the organism activates the unfolded protein response (UPR) to combat adverse external environments. Activated UPRs yield divergent results; on the one hand, cells undergoing short-term ERS can transport misfolded proteins to the ubiquitin–proteasome system (UPS) for degradation to promote cell survival (Hwang and Qi 2018); on the other hand, long-term ERS results in the UPR triggering CHOP or JNK to undergo apoptosis (Pu et al. 2023). However, the key factors causing the switch from pro-survival to pro-apoptotic effects of the UPR and whether the ERS-induced apoptotic pathway and UPS are involved in TP/LPS-associated hepatotoxicity remain unclear.

Apoptosis is also related to reactive oxygen species (ROS), and previous studies by our group have shown that TP can lead to oxidative stress via ROS accumulation, leading to hepatocyte apoptosis and hepatotoxicity (Hasnat et al. 2019, 2020). Numerous studies have reported on the crosstalk between ERS and ROS (Smyrnias 2021; Win et al. 2016; Zhang et al. 2002), but whether ERS can lead to apoptosis by inducing ROS production in TP/LPS-related liver injuries has yet to be validated.

In this study, we hypothesized that both the ERS-associated apoptotic pathway and the ERS-ROS-mediated apoptotic pathway are involved in TP/LPS-induced hepatotoxicity. Moreover, we investigated alterations in the UPS-ERS pathway under TP/LPS co-stimulation to determine the critical point that causes the pro-survival/pro-apoptotic transition in the UPR and the mechanism by which ERS regulates liver injury.

## Materials and methods

### Experimental drugs and reagents

TP (purity > 98%, HPLC, CAS number 38748–32-2) was bought from Sanling Biotechnology Company (Guilin, Guangxi, China). Lipopolysaccharide (LPS) (L2755), 4-phenylbutyric acid (4-PBA) (purity ≥ 99%, P21005), N-acetylcysteine (A7250, NAC) and butylated hydroxyanisole (B1253, BHA) were obtained from Sigma‒Aldrich (St. Louis, MO, USA). Suc-LLVY-AMC (CAS number 94367–21-2, HY-P1002) and MG-132 (CAS number 133407–82-6, HY-13259) were obtained from MedChemExpress Ltd. (Monmouth Junction, NJ, USA).

Serum Alanine aminotransferase (ALT) and aspartate aminotransferase (AST) were measured with kits from Nanjing Wittmann Biotechnology Co. Ltd. (Nanjing, China); reduced glutathione (GSH) assay kits, BCA Protein Assay Kits and Protein Lysis Buffer were purchased from Nanjing Beyotime Co. Ltd. (Nanjing, China); reagents (TRIzol, SYBR Green Master Mix reagent, and Reverse Transcription Kit) for qPCR were purchased from Vazyme Biotech Co., Ltd. (Nanjing, China); MEM was obtained from Gibco (Grand Island, NY, USA); CellROX Green Reagent and LipofectamineTM3000 Transfection Reagent were purchased from ThermoFisher Scientific (Waltham, MA, USA); CytoTox96® Non-Radioactive Cytotoxicity Assays (G1780) were obtained from Promega Corporation (Madison, Wisconsin, USA); siRNA Negative Control, siRNA-ATF4-688 and siRNA-ATF4-1132 were purchased from Shanghai Gemma Pharmaceuticals (Shanghai, China).

Antibodies against cleaved caspase-3 (9661), FLIP (56343s), ATF4 (11815s), eIF2α (9722.0), p-eIF2α (s51) (9721 s) and CHOP (2895) were obtained from Cell Signaling Technology (Danvers, MA, USA). Antibodies against GRP78 (n-20) (sc-1050) and GAPDH (sc-365062) were purchased from Santa Cruz Biotechnology (Dallas, TX, USA). Antibodies against cleaved PARP (ab32064) were purchased from Abcam (Waltham, MA, USA). Antibodies against β-actin (T0022) were obtained from Affinity Biosciences (Cincinnati, OH, USA). Alexa Fluor 647-conjugated goat anti-rabbit IgGs (H + L) were purchased from JAX. Goat anti-rabbit-HRP secondary antibodies (31460) and goat anti-mouse-HRP secondary antibodies (31430) were bought from Thermo Fisher Scientific (Waltham, MA, USA).

### Animals and experimental model

Female C57BL/6J mice (18-20g) with 6-8 weeks of age (mature stage in growth and development) were obtained from Vital River Laboratory Animal Technology (Beijing, China) for this research. The selection of experimental animals was based on our group’s former explorations of TP/LPS co-treatment due to the better sensitivity of female rats than male rats under exposure to the toxic dose of TP (Jiang et al. 2016). Mice were fed in plastic cages filled with corn cobs and freely access to water and food. All experimental procedures involving mice followed the guidelines of the Ethics Committee of China Pharmaceutical University and the Jiangsu Provincial Experimental Animal Management Committee (Approval No. 2022–06-014). Mice were acclimatized before the start of the experiments (1 week). The TP and LPS doses were selected based on articles published by our group (Yuan et al. 2019). After the administration of NAC (intraperitoneal injection), 4-PBA (intraperitoneal injection), TP (intragastric administration), LPS (intraperitoneal injection), or an equivalent volume of solvent (0.5% CMC-Na or PBS) to the control group (the dose administration is 10 mL/kg), the subsequent experiments were performed on collective liver samples and serum at the selective time points (3 h, 4 h, or 6 h). The choice of time point depended on the plasma concentration of NAC (maximum within 1-2 h, which means 2-3 h after TP/LPS treatment) (Tenório et al. 2021) and previous research on TP/LPS exposure (serious liver damage occurred in 6 h) (Yuan et al. 2020; Zhou et al. 2024). To be specific, after the first observation of ROS levels at 2 h after NAC administration, which means 3 h after TP/LPS treatment, there was no significant change. Therefore, we continued to explore the ROS level of TP/LPS treated for 4 h, at this time, a substantial increase was observed. Finally, the 6 h time point confirmed was based on the finding of our previous research that TP/LPS effect 6 h can cause serious liver damage.

To investigate whether TP pretreatment at different time points increased the sensitivity of the liver to LPS stimulation and to observe oxidative stress (OS) and apoptosis, 90 mice were equally divided into 15 groups; each mouse was given a single dose of 500 μg/kg of TP or the same volume of CMC-Na (0.5%), and 2 h later, 0.1 mg/kg of LPS or PBS was injected. One hour later, 300 mg/kg of NAC or an equal volume of PBS was injected intraperitoneally. Mice were sacrificed at 3, 4, and 6 h accordingly after LPS injection.

To demonstrate the protective effect of 4-PBA and the role of ERS in TP/LPS-associated liver damage, an additional 24 female C57BL/6J mice were equally divided into 4 groups. 400 mg/kg 4-PBA or PBS were injected intraperitoneally into Mice; 1 h later, they were gavaged with TP (500 μg/kg) or the same volume of CMC-Na (0.5%); and 2 h later, 0.1 mg/kg LPS or PBS were injected intraperitoneally into aforementioned mice. Sacrifices were performed at 6 h after the injection of LPS.

### Serum biochemistry analysis

The serum was collected at 4°C by centrifugation (1000 × g) for 15 min, and the levels of serum ALT and AST levels were measured at 340 nm. All assays followed the instructions of the manufacturer.

### Measurement of GSH

Plasma samples were obtained by centrifuging (600 × g, 10 min, 4°C) and adding protein remover M (50 μL). After centrifugation (10,000 × g, 10 min, 4°C), the supernatant was collected for the assay based on the instructions of the manufacturers, and the absorbance was tested at 412 nm.

### ROS assay

ROS levels in fresh liver tissue (10 mg) or cells were assayed using DCFH-DA or Cell ROX Green Reagent, and all assay procedures followed the manufacturer’s instructions. Detection was performed at a VARIOSKAN LUX multifunctional enzyme marker (Thermo Fisher Scientific, USA) (an excitation wavelength: 488 nm; an emission wavelength: 525 nm), and fluorescence images were captured through an FV-3000 (Olympus, TKY, Japan).

### Histopathological examination

Fresh mice livers were fixed, dehydrated, paraffin-embedded, and were finally sectioned (3-5 μm). Hematoxylin and eosin (H&E) stainings were employed for the observation of the liver samples’ histopathological and morphological changes.

### Terminal deoxynucleotidyl transferase dUTP nick-end labeling (TUNEL) assay

The apoptosis levels of liver tissue were detected by the TUNEL kits (Servicebio, Wuhan, China). After deparaffinization and hydration of paraffin sections, bovine serum albumin (3%) and normal calf serum (20%) were mixed to block nonspecific reactions and then added the TUNEL working solution dropwise. Images were cuptured under the microscope.

### Cell culture and cell viability assays

AML12 cells (the National Collection of Authenticated Cell Cultures, Shanghai, China) were co-cultured with the CM-0602 complete medium (Procell Life Science & Technology Co., Ltd., China) in a humidified atmosphere of 5% CO_2_ at 37°C.

Cell viability was determined with a CCK8 assay in 96-well plates, mainly used to determine the drug concentration. Upon culturing AML12 cells for an appointed time, the supernatants were discarded, and according to the manufacturer’s instructions, the corresponding proteins were detected.

Lactate dehydrogenase (LDH) assays, which detect the damaged cells’ release of lactate dehydrogenase via CytoTox 96^®^ Non-Radioactive Cytotoxicity Assay Kit., were to examine cell toxicity.

### Proteasome activity assay

After protein extraction from fresh liver tissues (20 mg) or cells, the fluorescent signal values were detected every 15 min for at least 2 h after adding the AMC fluorescent substrate (MedChemExpress, NJ, USA) with a VARIOSKAN LUX multifunctional enzyme marker (excitation wavelength: 380 nm; emission wavelength: 460 nm). All procedures followed the manufacturer’s instructions.

### Immunocytochemistry

AML12 cells were treated with the appropriate drugs for the indicated times, fixed with 10% formaldehyde for 15 min, washed with PBS 3 times (5 min each), permeabilized with Triton X-100 (0.1%) for 15 min, and subsequently washed in the above same way. The fixed cells were then incubated with an anti-ATF4 primary antibodies (4°C, overnight), following with the incubation of an Alexa Fluor 647 secondary antibodies (room temperature, 1 h). DAPI (Beyotime Biotechnology, Shanghai, China) labeled the nuclei for a quarter of an hour, and the images of fluorescence were captured using an FV-3000.

### Western blot analysis

Total proteins were extracted from the liver samples or AML12 cells and were subsequently quantified by BCA protein assay kits. The proteins were transferred onto a PVDF membrane after the separation via SDS‒PAGE, which was blocked for 1 h with 5% BSA (room temperature) and then incubated overnight with specific antibodies at 4°C. The membranes were then incubated at room temperature with appropriate secondary antibodies for 1 h. Enhanced chemiluminescence (ECL) was employed for imaging in the gel imaging system.

### RNA extraction and qPCR

Total mRNAs were extracted from liver samples or AML12 cells using TRIzol reagent. After the quantification via Nanodrop 2000 (Thermo Fisher Scientific, USA), 1 μg/μL of RNAs were reverse transcribed to cDNAs. AceQ^®^ SYBR Green Master Mix (High ROX Premixed) and Applied Biosystems StepOne™ real-time quantitative PCR (Bio-Rad Laboratories, Hercules, CA, USA) were used. The primer sequences are listed in Table 1, and the amount of cDNAs was standardized by GAPDH.

**Table 1:** Sequences of the target gene siRNAs

Gene	Gene sequence (5'-3')
Negative control sense	UUCUCCGAACGUGUCACGUTT
Negative control antisense	ACGUGACACGUUCGGAGAATT
ATF4-Mus-687 sense	GUCUCUUAGAUGACUAUCUTT
ATF4-Mus-687 antisense	AGAUAGUCAUCUAAGAGACTT
ATF4-Mus-963 sense	CUCUAGCCCAAGAGACUAATT
ATF4-Mus-963 antisense	UUAGUCUCUUGGACUAGAGTT
ATF4-Mus-1132 sense	GCUAGGCAGUGAAGUUGAUTT
ATF4-Mus-1132 antisense	AUCAACUUCACUGCCUAGCTT

### Cell transfection of siRNAs

siRNAs were synthesized by GenePharma, and the sequences of siRNAs are listed in Table 1. The 20 μM primer solution was obtained by dissolving siRNAs in DEPC water. AML12 cells (at 40%-60% cell density) cultured in 12-well plates were transfected with siRNAs (100 μL MEM mixed with 5 μL primer solution) along with 5 μL Lipofectamine 3000 mixed with 100 μL MEM according to the manufacturer’s protocols. Let above stand at room temperature for 15 min. After 8 h of transfection, the original medium was discarded, and fresh MEM was added to it in the plates for another 24 h before co-administration of TP (50 nM) and TNF-α (50 ng/mL). The cells were collected 24 h after the administration of TNF-α for the desired experiments.

### Quantification and Statistical analysis

All the experimental data were presented in the form of the means ± SEMs and the analysis was performed by GraphPad Prism 8.0. The results of liver sections were imaged and analyzed using Image J and the proportion of necrotic area was evaluated by Photoshop (Version 2024) for quantification. Group differences evaluation was conducted via One-way analysis of variance (ANOVA) and Tukey’s multiple comparison test. For all analyses, P value < 0.05 indicated statistical significance. * *P* < 0.05, ** *P* < 0.01, *** *P* < 0.001, which were mentioned in all legends of the figures.

## Results

### TP/LPS induced acute hepatotoxicity in mice by triggering ROS

**Fig. 1:**
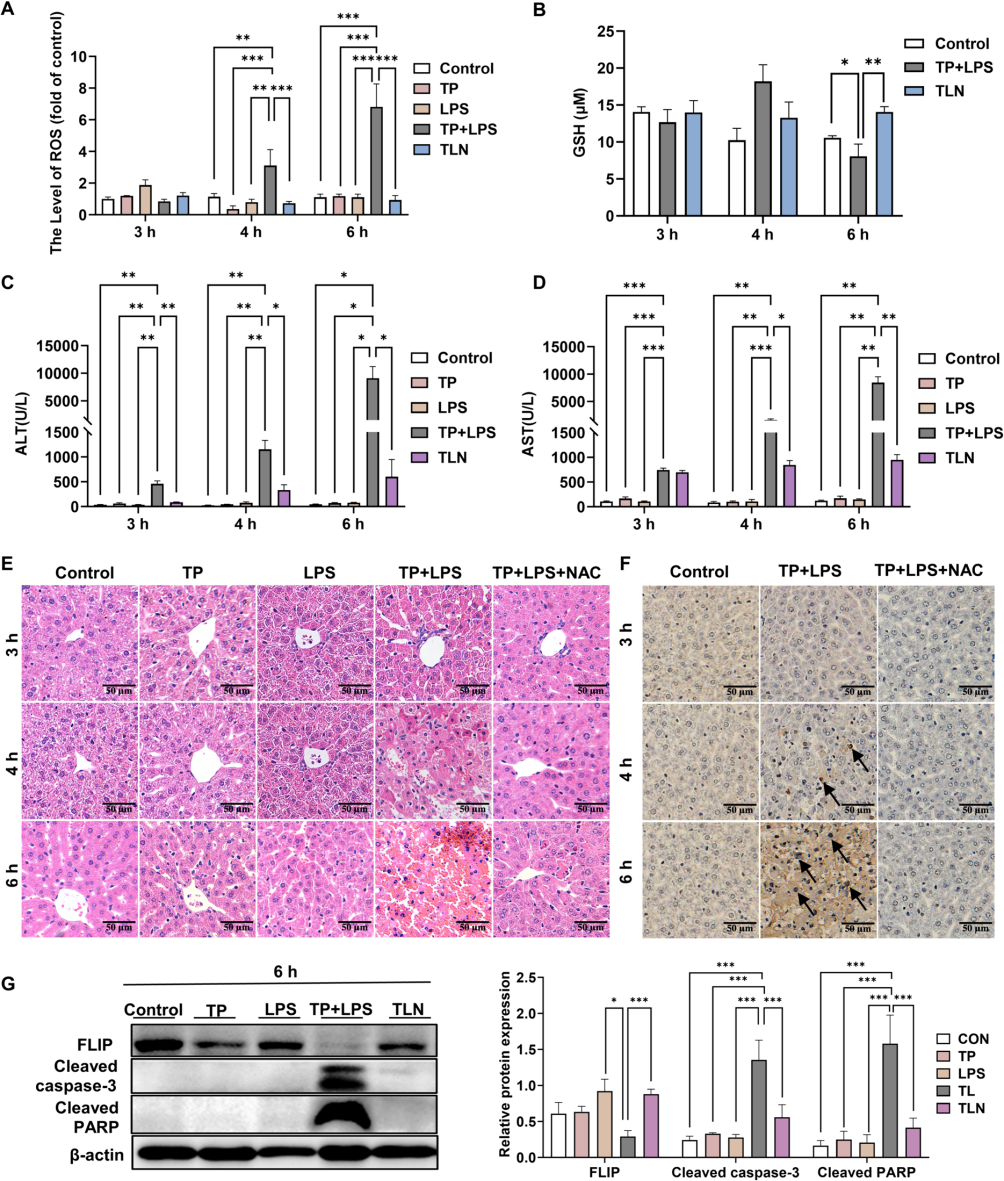
TP/LPS activated the ROS-dependent apoptotic pathway. (**A**) ROS levels in mice at different times. (**B**) GSH levels in mice at different time points. (**C**-**D**) Serum ALT and AST in mice treated with TP, LPS, NAC or both at different times. (**E**) H&E-staining images of mice liver tissue Sects. (400 × , Scale bar = 20 μm). (**F**) TUNEL staining images of mice liver tissue Sects. (200 × , Scale bar = 50 μm). (**G**) At 6 h, representative illustrative Western blot images and corresponding relative intensities of the protein bands of apoptosis-related proteins, with β-actin acting as the loading control. The results are presented in the form of means ± SEMs. *P < 0.05, **P < 0.01, ***P < 0.001, *n* = 6

To investigate whether TP/LPS can trigger ROS and to explore how TP/LPS-induced liver damage occurred during three selective time points (3 h, 4 h, and 6 h; the selection of the time points was mentioned in Method section) in female C57BL/6 mice, we administered the antioxidant NAC (300 mg/kg) after modeling and handled the samples in these indicated time. The results for the oxidative stress-related indicators ROS and GSH showed that after TP/LPS treatment for 4 h, the ROS levels increased significantly (Fig. 1A), and at 6 h, the GSH levels decreased significantly (Fig. 1B). This phenomenon can be reversed by administering NAC. Elevated ALT and AST levels were observed with prolonged exposure to TP/LPS, which emerged early at 3 h; on the contrary, no significant changes existed in TP or LPS alone groups (Fig. 1C-D). Previous research in our laboratory has shown that at 1 h many inflammatory factors were released, and no liver injury was observed; At 2-3 h, the TNF-α level returned to a normal level (the early stage); at 4-5 h, the inflammatory factor downstream pathway began to change (the middle stage); and at 5-8 h, severe liver damage occurred (the late stage) (Yuan et al. 2020). Similarly, in this research, histopathologic assessment revealed nuclear rupture at 4 h after TP/LPS coadministration, and at 6 h, distinct morphological abnormalities were observed in the liver. In contrast, neither TP nor LPS alone affected normal liver histology (Fig. 1E; Fig. S2A). Moreover, TUNEL staining revealed TUNEL-positive cells at 4 h, and at 6 h, increased TUNEL-positive areas were observed in the liver (Fig. 1F; Fig. S2B). After confirming that ROS and apoptosis are involved in TP/LPS-induced hepatotoxicity, we administered NAC and tested apoptosis-related proteins. At 6 h postinjection of LPS, the peak of induced liver damage in this model, we observed elevated cleaved caspase-3 and cleaved PARP protein levels associated with decreased FLIP, which were significantly reversed by NAC compared with those in the controls (Fig. 1G). The results above indicated that NAC could inhibit TP/LPS-induced apoptosis.

### The ERS-specific inhibitor 4-PBA protected against TP/LPS-related apoptosis and the oxidative stress within hepatocytes

**Fig. 2:**
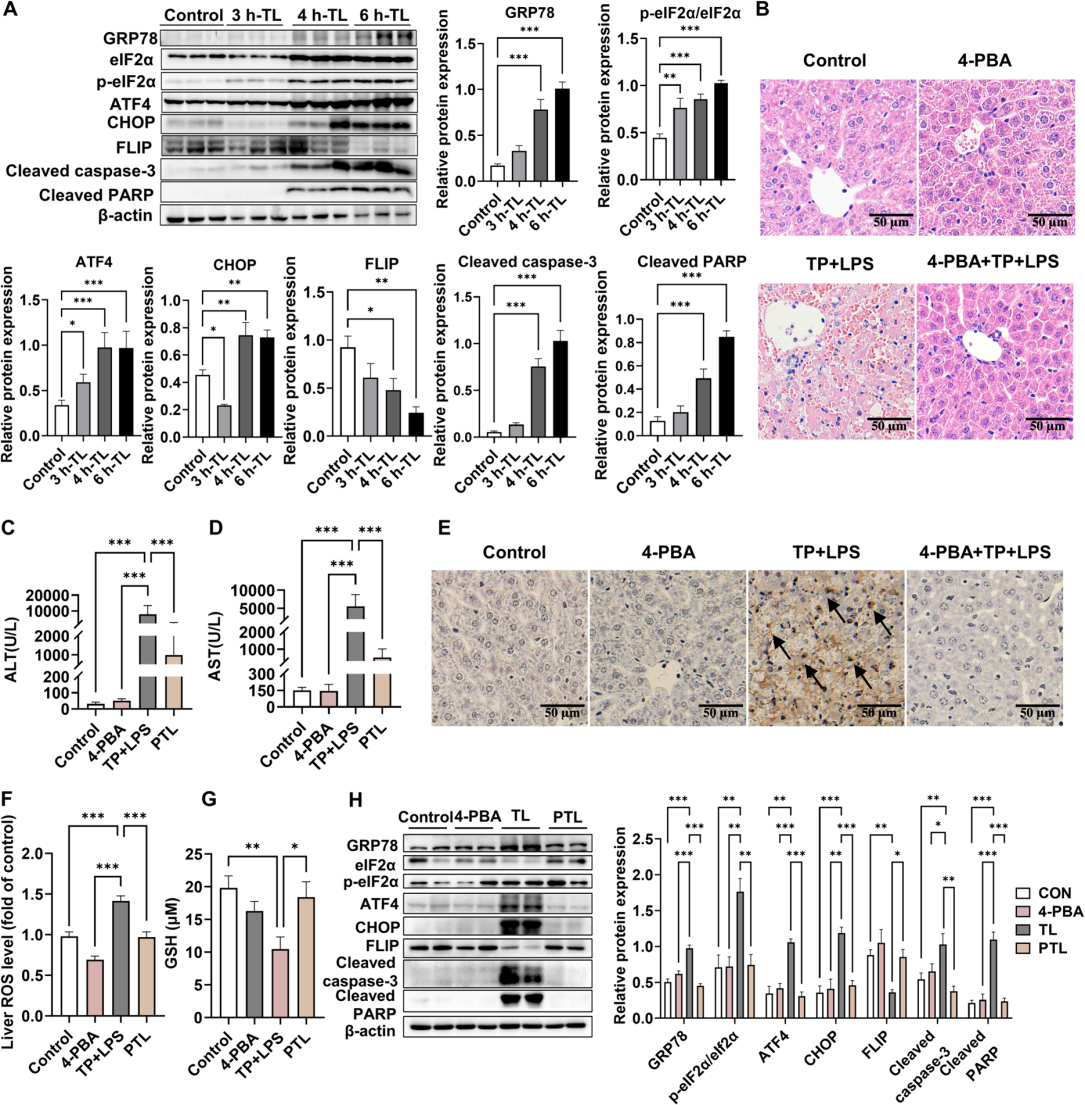
Effects of 4-PBA administration on hepatotoxicity triggered by TP/LPS cotreatment. (**A**) Representative illustrative Western blot images at different times and the relative intensity of the protein bands concerning ERS-related and apoptosis-related proteins, with β-actin acting as the loading control. (**B**) H&E-staining images of mice liver tissue Sects. (400 × , Scale bar = 20 μm). (**C**-**D**) Alterations in the blood biochemical parameters (serum ALT and AST) of the mice. (**E**) TUNEL staining images of mice liver tissue Sects. (200 × , Scale bar = 50 μm). (**F**-**G**) Effect of 4-PBA preadministration on ROS and GSH levels in liver tissues. (**H**) Representative illustrative Western blot images and relative intensities of protein bands corresponding to the GRP78-ATF4-CHOP signaling pathway and cleaved caspase-3, cleaved PARP, and FLIP in vivo normalized to that of β-actin. The results are presented in the form of the means ± SEMs. *P < 0.05, **P < 0.01, ***P < 0.001, *n* = 6

Numerous studies have reported that ERS and its mediation of apoptosis and oxidative stress result in the development of various liver diseases. As expected, we found that TP/LPS stimulation increased GRP78, ATF4, p-eIF2α/eIF2α, and cleaved caspase-3 and PARP expression in a time-dependent manner, accompanied by decreased FLIP protein levels (Fig. 2A). To explore the involvement of ERS in TP/LPS-induced hepatotoxicity and to explore potential protective strategies, we pretreated mice with 400 mg/kg 4-PBA, an ERS-specific inhibitor, and sacrificed the mice 6 h after LPS injection. Mice in the TP/LPS cotreatment group exhibited obvious edema, hepatic vacuolar degeneration, inflammatory cell infiltration and liver histomorphology accumulation, which was reversed by pretreatment with 4-PBA, and no hepatotoxicity was observed with 4-PBA alone (Fig. 2B; Fig. S3A). In addition, preadministration of 4-PBA significantly reduced the increase in ALT and AST levels induced by TP/LPS (Fig. 2C-D). The TUNEL results showed that preadministration of 4-PBA downregulated TP/LPS-induced hepatoapoptosis (Fig. 2E; Fig. S3B). We also found that TP/LPS induced ROS accumulation and significantly reduced GSH levels, which could be reversed by preadministration of 4-PBA (Fig. 2F-G). Furthermore, the increase in GRP78, ATF4, p-eIF2α/eIF2α, CHOP, cleaved caspase-3 and cleaved PARP, and the decrease in FLIP protein expression induced by TP/LPS were also significantly reversed by 4-PBA administration (Fig. 2H). These results showed that pretreatment with 4-PBA could attenuate TP/LPS-related liver damage in C57BL/6J mice.

#### PBA improved hepatocyte apoptosis under TP/TNF-α cotreatment

**Fig. 3:**
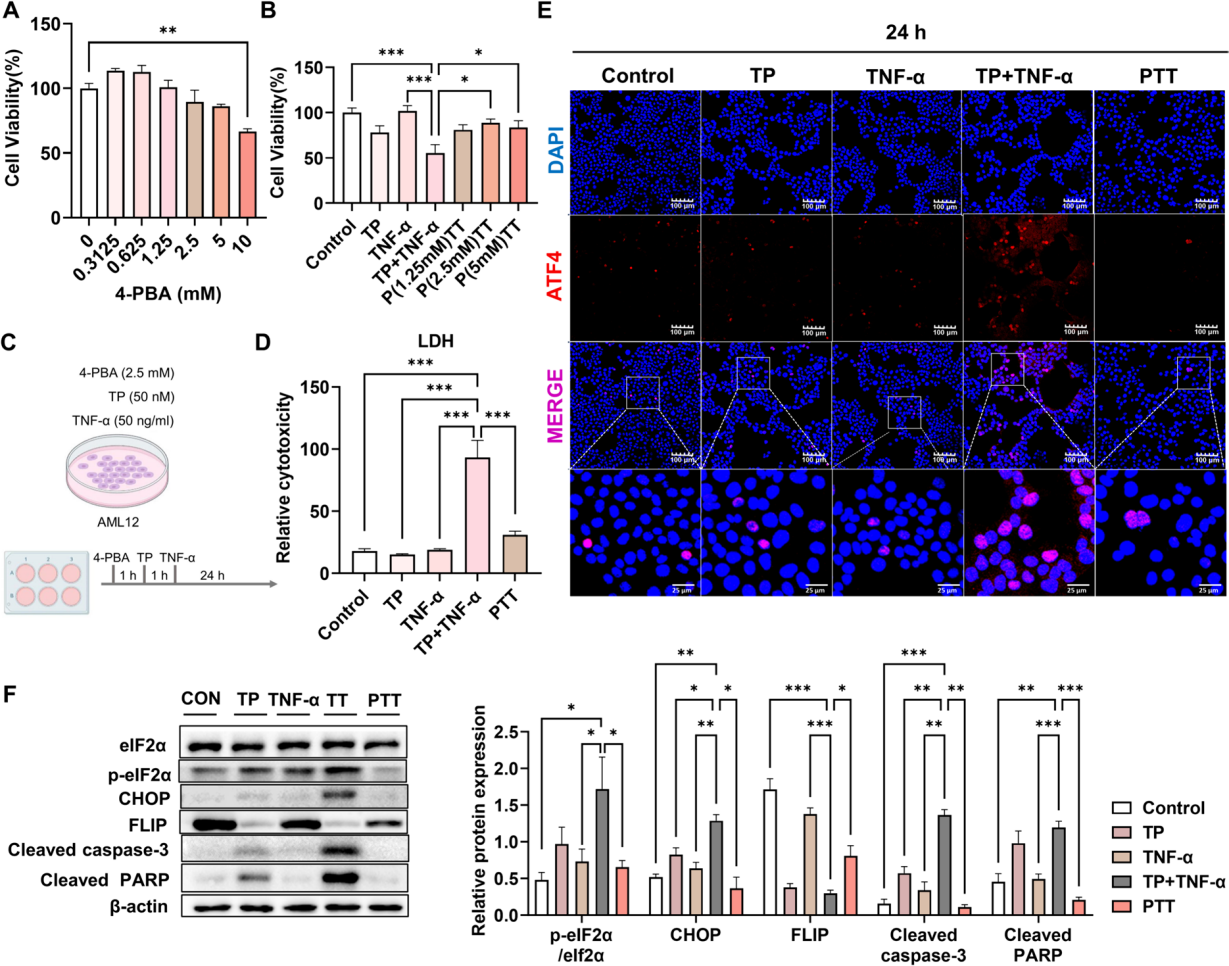
Effects of 4-PBA administration on hepatotoxicity induced by TP/TNF-α cotreatment. (**A**-**B**) The optimal dose of 4-PBA was confirmed with a CCK-8 assay. (**C**) Schematic representation of the experimental procedure. (**D**-**E**) AML12 cells were treated with 4-PBA (2.5 mM) plus TP (50 nM)/TNF-α (50 ng/mL) and were collected at 24 h after TNF-α administration for LDH and immunofluorescence assays, respectively. (**F**) Representative illustrative Western blot images and relative intensities of the protein bands concerning eIF2α, p-eIF2α, CHOP, FLIP, cleaved caspase-3 and cleaved PARP, with β-actin acting as the loading control. The results are presented in the form of the means ± SEMs. *P < 0.05, **P < 0.01, ***P < 0.001, *n* = 3

To further verify the efficacy of 4-PBA in vitro, we used TNF-α instead of LPS as a stimulant, for the reason that Toll-like receptor 4 is lowly expressed within hepatocytes and TNF-α regulated NF-κB activation and cell death, while decreased level of TNF-α was associated with counteracting TP-induced hepatotoxic reactions concerning apoptosis and necroptosis (Wang et al. 2019; Yuan et al. 2020). We initially determined the optimal cellular concentration of 4-PBA (2.5 mM) using a CCK-8 assay (Fig. 3A-B). The flowchart of cellular administration is shown in Fig. 3C. TP/TNF-α cotreatment significantly increased LDH release, which was reversed by pretreatment with 2.5 mM 4-PBA (Fig. 3D). As anticipated, preadministration of 4-PBA reduced ATF4 levels, decreased the expression of ERS-related proteins, and inhibited TP/TNF-α-induced apoptosis (Fig. 3E-F; Fig. S4A). These findings collectively indicated that 4-PBA protected against TP/TNF-α-induced hepatotoxicity via inhibiting ERS-dependent apoptosis.

### Interference with ATF4 alleviated cell apoptosis by inhibiting ROS accumulation

**Fig. 4:**
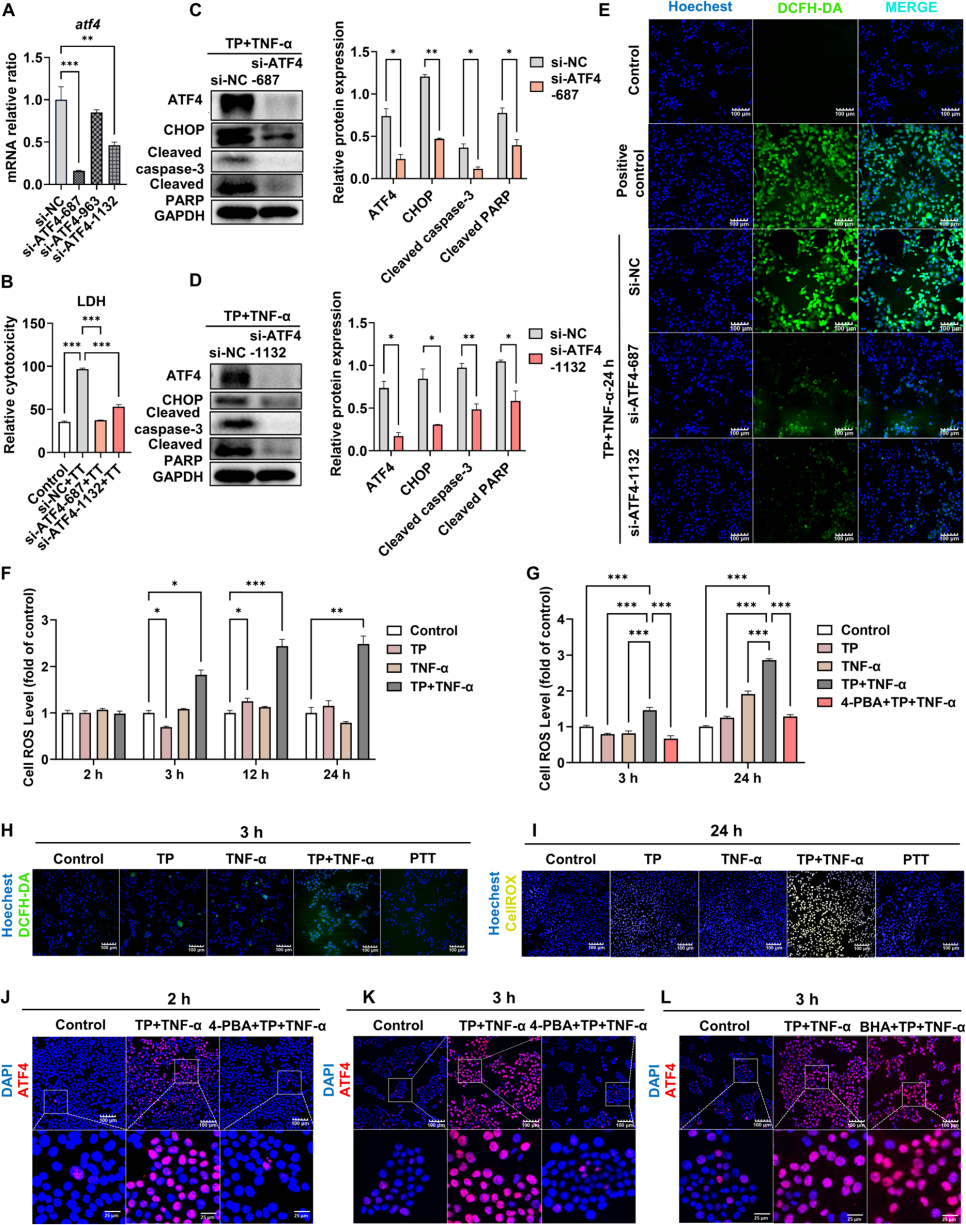
Interference with ATF4 alleviated cell apoptosis by inhibiting ROS accumulation. (**A**) Verification of the knockdown efficiency of ATF4 in AML12 cells. (**B**) Relative LDH leakage of AML12 cells treated with si-NC or si-ATF4 plus TP/TNF-α for 24 h. (**C**-**D**) Treated AML12 cells were collected at 24 h after TNF-α administration. Representative illustrative Western blot images and relative intensities of the protein bands of ATF4, CHOP, cleaved caspase-3, and cleaved PARP are shown, with GAPDH acting as the loading control. (**E**) Treated AML12 cells were collected at 24 h after TNF-α treatment, and ROS levels were detected by DCFH-DA. (**F**) Treated AML12 cells were collected at 2, 3, 12 and 24 h after TNF-α administration for ROS detection by DCFH-DA. (**G**) AML12 cells were treated with 4-PBA (2.5 mM) plus TP/TNF-α and were collected 3 h and 24 h after TNF-α application for ROS detection by DCFH-DA. (**H**-**I**) Treated AML12 cells were collected 3 h and 24 h after TNF-α treatment to detect ROS levels. (**J**) AML12 cells were treated with 4-PBA plus TP/TNF-α and collected 2 h after TNF-α treatment for detection of ATF4 expression. (**K**-**L**) AML12 cells were treated with 4-PBA or BHA (100 μM) plus TP/TNF-α and were at collected 3 h after TNF-α administration for detection of ATF4 expression. The results are presented in the form of the means ± SEMs. *P < 0.05, **P < 0.01, ***P < 0.001, *n* = 3

According to the above findings, TP/LPS-induced ERS activated the GRP78-ATF4-CHOP pathway. Given that ATF4 targets CHOP, an important regulator of ERS-mediated apoptosis, we used cells with small interference in subsequent investigations to explore the potential role of ATF4 in TP/LPS-related hepatotoxicity. ATF4 expression was significantly decreased in cells transfected with siATF4-687 or siATF4-1132, underscoring the effectiveness of the knockdown procedure (Fig. 4A). Furthermore, we observed that siATF4-687 and siATF4-1132 mitigated the cytotoxic effects induced by TP/TNF-α (Fig. 4B), markedly suppressing CHOP expression and apoptosis (Fig. 4C-D). Notably, compared to cells transfected with NC, hepatocytes transfected with siATF4 exhibited protective effects against TP/TNF-α-induced ROS release at 24 h (Fig. 4E; Fig. S5A).

Evidence suggests that mitochondria and the ER can interact through mitochondria-associated membranes (MAMs) (Area-Gomez and Schon 2016; Aufschnaiter et al. 2017). This insight prompted us to focus on the intricate relationship between oxidative stress and ERS. At the cellular level, we detected significant changes in ROS levels at 3 h, which persisted until 24 h after TP/TNF-α exposure (Fig. 4F). Moreover, pretreatment with 4-PBA significantly mitigated the abnormally elevated ROS levels (Fig. 4G, I; Fig. S5C). Next, we examined the time points of ROS and ERS production by staining for ATF4 and ROS. The results showed that ERS occurred at 2 h when TP/TNF-α affected AML12 cells (Fig. 4J; Fig. S5D), but there were no changes in ROS levels at this time point (Fig. 4F). In contrast, at 3 h, ERS was significantly induced, and preadministration of 4-PBA significantly inhibited ERS and ROS production (Fig. 4H, K; Fig. S5B, E). However, preadministration of 100 μM butylated hydroxyanisole (BHA), an antioxidant that scavenges ROS by donating labile hydrogen to oxygen free radicals (Festjens et al. 2006), failed to decrease the number of ATF4-positive cells (Fig. 4L; Fig. S5F). These findings suggested that TP/LPS can induce ROS accumulation, leading to apoptosis by upregulating ATF4, and that ATF4 may serve as a crucial regulator in determining the survival or death of hepatocytes in the context of TP/LPS-induced conditions.

### TP/LPS inhibits proteasome activity, inducing ERS-mediated hepatotoxicity

**Fig. 5:**
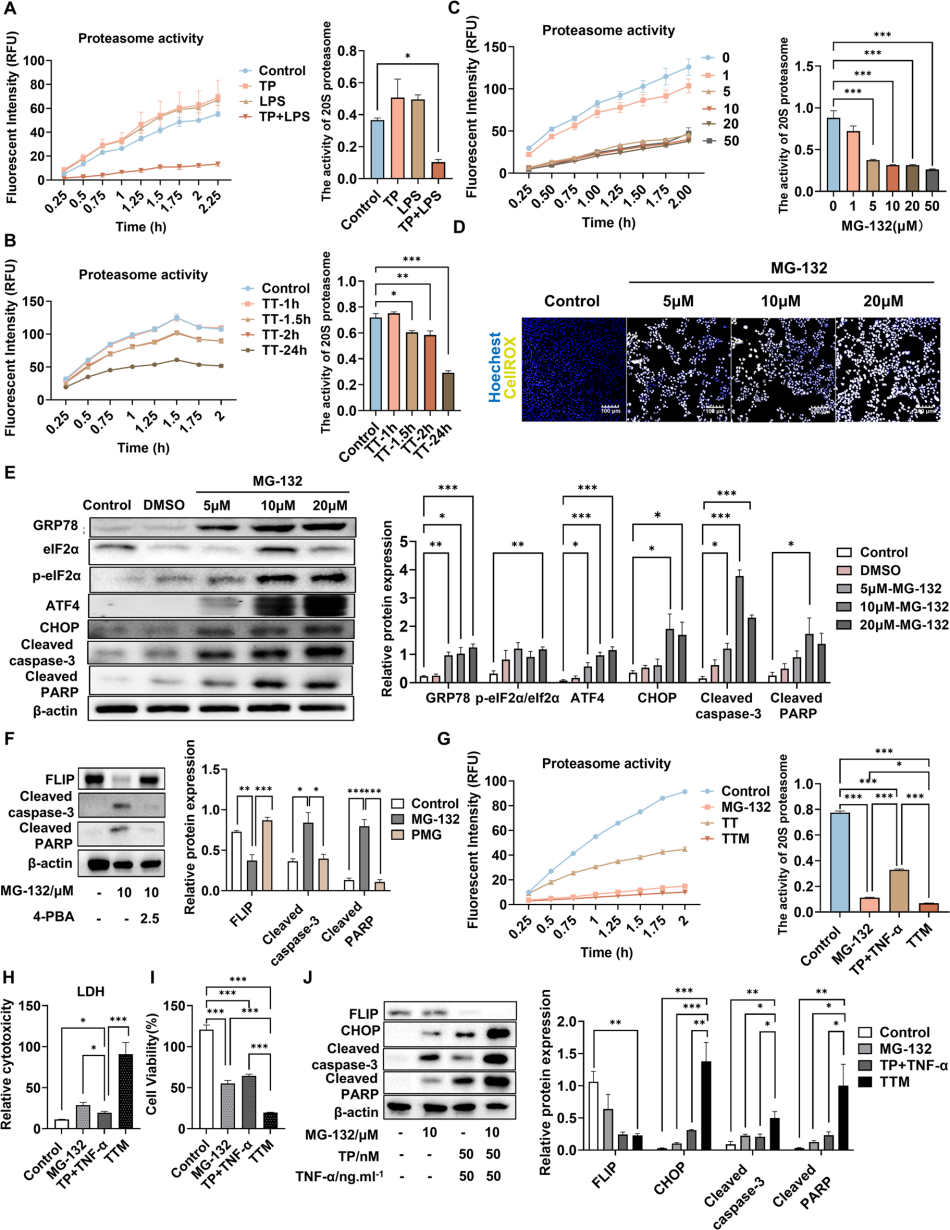
Inhibition of proteasome activity was responsible for the production of ERS induced via TP/LPS or TP/TNF-α cotreatment. (**A**) Changes in liver proteasome activity in vivo at 6 h after LPS administration (*n* = 6). (**B**) Changes in proteasome activity in AML12 cells treated with TP/TNF-α were assessed at 1, 1.5, 2 and 24 h after TNF-α administration. (**C**) Changes in liver proteasome activity within AML12 cells treated with different concentrations of MG-132. (**D**) AML12 cells from different groups were collected, and ROS levels were detected with CellROX Green Reagent. (**E**) AML12 cells were treated with 5, 10 or 20 μM MG-132 for 24 h and collected. The protein levels of GRP78, eIF2α, p-eIF2α, ATF4, CHOP, cleaved caspase-3, and cleaved PARP in vitro were determined by western blot analysis and normalized to that of β-actin. (**F**) AML12 cells were treated with 4-PBA (2.5 mM), MG-132 (10 μM) or both for 24 h and were collected. The representative illustrative Western blot images and relative intensities of the FLIP, cleaved caspase-3 and cleaved PARP protein bands are shown, with β-actin serving as the loading control (*n* = 3). (**G**) Alterations in proteasome activity in AML12 cells in different groups. (**H**-**I**) LDH and CCK8 assays for detecting the cytotoxicity of different groups of AML12 cells. (**J**) AML12 cells treated with TP, TNF-α, MG-132 or both were collected 24 h after MG-132 treatment. Representative illustrative Western blot images and relative intensities of the FLIP, CHOP, and cleaved caspase-3 protein bands and of the cleaved PARP band, with β-actin acting as the loading control. The results are presented in the form of the means ± SEMs. *P < 0.05, **P < 0.01, ***P < 0.001, *n* = 3

ER-associated protein degradation (ERAD) has been reported to eliminate misfolded proteins produced by ERS (Hwang and Qi 2018), suggesting that proteasome activity is helpful for alleviating ERS. To investigate our hypothesis that TP/LPS induces ERS by inhibiting proteasome activity, we assessed proteasome activity in tissues and cells using an AMC fluorescent substrate probe. The results revealed that the coadministration of TP/LPS significantly inhibited proteasome activity; at the same time, a similar effect was not observed with the individual administration of TP or LPS alone (Fig. 5A). Furthermore, in AML12 cells, proteasome activity was suppressed significantly at 1.5 h, 2 h, and 24 h after TP/TNF-α cotreatment (Fig. 5B).

To further illustrate the relationship between proteasome activity and ERS, proteasome activity assays were performed in vitro via the administration of MG-132 (1, 5, 10, 20 and 50 μM), a proteasome activity inhibitor. The result demonstrated the inhibitory effects of MG-132 in a general dose-dependent manner in which administration of 5 μM MG-132 can inhibit the proteasome activity of AML12 cells. (Fig. 5C). Furthermore, MG-132 resulted in exacerbated ROS accumulation and increase of GRP78, ATF4, CHOP, p-eIF2α/eIF2α, cleaved caspase-3 and cleaved PARP protein levels, which suggested that the impaired degradation ability of proteasome leads to the accumulation of GRP78, thereby triggering ERS. (Fig. 5D-E; Fig. S6A). Remarkably, preadministration of 4-PBA could downregulate the expression of the above-mentioned apoptosis-related proteins, further indicating the protective effect of 4-PBA (Fig. 5F). To further elucidate whether TP/LPS or TP/TNF-α induces ERS by inhibiting proteasome activity, we co-administered MG-132 with TP/TNF-α. As shown in Fig. 5G, the proteasome activity was further inhibited, and exacerbated cytotoxicity occurred (Fig. 5H-I). Moreover, along with the increase in the CHOP protein level upon MG-132 and TP/TNF-α exposure, the levels of apoptotic proteins, including cleaved caspase-3 and cleaved PARP, also increased, while the expression of pro-survival proteins, such as FLIP, decreased (Fig. 5J). These results suggested that TP/LPS induced hepatotoxicity by activating the ERS-associated apoptotic pathway through the suppression of proteasome activity.

## Discussion

In this research, we explored the relationships among oxidative stress, ERS, and proteasome activity via detections during different time points, providing novel insights into the effect of proteasome activity in ERS-induced apoptosis and the role of ERS in ROS accumulation during TP/LPS-induced hepatotoxicity. We demonstrated the involvement of ATF4 in ERS-induced ROS accumulation, as indicated by a marked reduction in ROS levels when ATF4 was knocked down. These findings suggested that maintaining proteasome activity might be a therapeutic strategy for improving cell survival and death in patients with TP/LPS-induced hepatotoxicity.

Cell death is crucial because it eliminates damaged cells to maintain tissue homeostasis within healthy tissues, but it is also responsible for pathologies that occur leading to chemically induced toxicity. Our group’s previous findings indicated that liver injury caused by TP + LPS involved apoptosis and necroptosis (Yuan et al. 2020, 2019), and recent research indicated that TP-induced injuries were characterized by apoptosis rather than necroptosis via the results of metabolomics and RNA-seq examination (Zhao et al. 2020). So in this study, we tended to focus on the potential mechanism under TP/LPS model concerning apoptosis. Apoptosis, a programmed cell death process (immunologically and highly coordinated), was commonly reported to play a vital role in TP-associated hepatotoxicity (Bertheloot et al. 2021; Guicciardi et al. 2013; Hu et al. 2022). In accord with previous research, we also observed that the toxic effect of TP/LPS on the liver histologically manifested as hepatocyte apoptosis (significant changes appeared at 4 h), along with increased ERS-related apoptosis protein expression levels of GRP78, ATF4, p-eIF2α/eIF2α, CHOP (mostly changed significantly at 3 h, expect GRP78), cleaved caspase-3 and cleaved PARP (changes appeared at 4 h). These findings indicated that ERS-induced apoptosis is related to TP/LPS-induced hepatotoxicity. What’s more, our study surprisingly revealed that MG-132, a proteasome inhibitor, further aggravated TP/TNF-α induced-cytotoxicity, and inhibited the expression of FLIP. Considering that the apoptosis inhibitory protein FLIP is mainly degraded by the UPS pathway and that the redox alterations profoundly affect the proteasome system (Ciechanover and Schwartz 1998; Lecker et al. 2006; Lefaki et al. 2017), we hypothesized that MG-132 induces the accumulation of ROS by inhibiting proteasome activity and that ROS can intervene in the ubiquitylation of FLIP, possibly accelerating its ubiquitylation, thereby inhibiting its expression.

In our previous research, we demonstrated that a single administration of a high dose of TP did not induce significant hepatic damage (Wang et al. 2014a, b; Wang et al. 2014a, b; Wang et al. 2016), which is inconsistent with clinical reports concerning TP-containing drugs can lead to severe liver injury. In light of this, our research group adopted a strategy of TP pretreatment followed by the administration of a nontoxic dose of LPS to mimic potential clinical adverse hepatic effects associated with TP (Yuan et al. 2020). In this study, C57BL/6 J female mice were chosen due to their hypersensitivity to TP-induced hepatotoxicity (Jiang et al. 2016). We found that with the prolonged duration of action of TP/LPS, mice showed dramatic upregulation of aminotransferase levels, significant alterations in liver morphology (significant changes appeared at 3 h), and imbalances in pro-survival/pro-apoptotic protein expression, which were not observed when TP or LPS was administered alone. Furthermore, the above alterations could be reversed by the addition of NAC, indicating the crucial role of oxidative stress in TP/LPS-induced liver injury, which represents a novel approach to mitigate the damaging effects of TP/LPS (Fig. 1).

There exists an intricate interaction between ERS and oxidative stress. On the one hand, ERS can be triggered by ROS accumulation (Oakes and Papa 2015). On the other hand, ERS can cause impaired ER lipid metabolism with consequent generation of large amounts of ROS (Zhang et al. 2019). Both ERS and ROS are reportedly involved in many liver injury diseases (Pu et al. 2023; Wang and Kaufman 2016), similarly occurring in our TP/LPS or TP/TNF-α models. In vivo, the levels of ROS changed significantly at 4 h, while the most tested ERS-associated protein levels changed at 3 h. By examining the time points of ROS and ERS production in AML 12 cells through corresponding probes and observing that 4-PBA (an ERS inhibitor) could inhibit abnormally elevated ROS levels and that BHA (an antioxygen) failed to relieve ATF4-positive cells at 3 h, we proposed that the occurrence of ERS might be anterior to ROS production. It is worth noting that we don’t deny the possibility of subsequent crosstalk between ERS and ROS. In addition, we prefer to speculate that the generation of ERS also affected the blood biochemical parameters (ALT and AST) of livers, whose abnormalities appeared at 3 h, for the existing evidence that the inhibition of ERS could relieve liver damage with lower ALT/AST levels (Lu et al. 2022; Ruan et al. 2020). According to the significant increase in GRP78, ATP4, CHOP, p-eIF2α/eIF2α, cleaved caspase-3, and cleaved PARP protein expression compared with that in the control group, we focused on the GRP78-ATF4-CHOP pathway and identified ATF4 as an essential regulator of TP/LPS-related hepatotoxicity due to the fact that ATF4 targets CHOP and is associated closely with ERS-mediated apoptosis. To verify the contribution of ATF4 to TP/LPS-induced hepatotoxicity, we subjected ALM12 cells to siRNA-ATF4-678 and siRNA-ATF4-1132 knockdown, which ameliorated liver damage, suppressed the expression of CHOP and inhibited apoptosis and ROS accumulation. Our findings indicated that ERS, which involves the GRP78-ATF4-CHOP pathway, may induce apoptosis by inducing ROS accumulation, which was reversed by 4-PBA (Figs. 2-4).

During ERS, misfolded proteins are degraded via the ERAD or autophagy-lysosomal pathway, in which approximately 80%-90% of misfolded proteins are degraded through the ERAD (Ciechanover and Kwon 2015; Kwon and Ciechanover 2017). ERAD is a multistep process involving the recognition of ERAD substrates, retrotranslocation, ubiquitination, and proteasome mediation (Hwang and Qi 2018). The proteasome inhibitor MG-132 can reportedly induce ERS in alveolar epithelial cells (Gallastegui and Groll 2010), and TP inhibits proteasome chymotrypsin-like activity and promotes apoptosis in cultured PC-3 cells in a time- and dose-dependent manner (Lu et al. 2011). Here, we used MG-132 to explore the relationships among the proteasome pathway, ERS, and apoptosis upon TP/LPS exposure. The results suggest that disturbance of proteasome activity can result in hepatic oxidative stress responses and liver cascade injury, which are closely related to ERS-induced apoptosis (Fig. 5). Based on the results obtained, it is suggested that inhibition of proteasome activity could lead to the accumulation of GRP78 and trigger ERS. However, whether the proteasome system directly acts on ATF4 and how the ATF4 acts on proteasome activity upon TP exposure remain to be further explored. Briefly, further characterization of the crosstalk between the endomembrane system and oxidative stress is likely to expand novel mechanistic information on TP/LPS-induced hepatotoxicity.

## Conclusions

In summary, our findings reveal that TP/LPS induces ERS-related apoptosis and hepatic oxidative stress by inhibiting proteasome activity. Mechanistically, TP/LPS-mediated liver injury may be attributed to the activation of GRP78-ATF4-CHOP axle or through ATF4-ROS interaction. Collectively, we provide valuable insights for guiding the development of new treatments, particularly in the context of TP poisoning, suggesting that ATF4 may be a key regulator in the survival or death of hepatocytes upon TP/LPS co-exposure.

## Supplementary Information

Below is the link to the electronic supplementary material.

Supplementary file1 (DOCX 1271 KB)

## Data Availability

No datasets were generated or analysed during the current study.

## Abbreviations

ALT: Alanine aminotransferase
APAP: Acetaminophen
AST: Aspartate aminotransferase
ATF4: Activating transcription factor 4
ATF6: Activating transcription factor 6
BHA: Butylated hydroxyanisole
CHOP: CCAAT/enhancer-binding protein homologous protein
eIF2α: Eukaryotic initiation factor-2α
ER: Endoplasmic reticulum
ERAD: ER-associated protein degradation
ERS: Endoplasmic reticulum stress
FLIP: FLICE inhibitory-protein
GRP78: Glucose regulated protein 78
GSH: Reduced glutathione
IRE1: Inositol-requiring enzyme 1
JNK: C-Jun N-terminal kinase
LDH: Lactate dehydrogenase
LPS: Lipopolysaccharide
MAMs: Mitochondria-associated membranes
OS: Oxidative stress
p-eIF2α: Eukaryotic initiation factor-2α phosphorylation
PERK: Protein kinase R-like ER kinase
ROS: Reactive oxygen species
TNF-α: Tumor necrosis factor-α
TP: Triptolide
TWHF: Tripterygium wilfordii Hook. f.
TUNEL: Terminal Deoxynucleotidyl Transferase dUTP Nick-End Labeling
UPR: Unfolded protein response
UPS: Ubiquitin proteasome system
4-PBA: 4- Phenylbutanoic acid
