# Supplementary material for: Proteasome activity inhibition mediates endoplasmic reticulum stress-apoptosis in triptolide/lipopolysaccharide-induced hepatotoxicity
Source: Cell Biol Toxicol. 2024 Jul 29;40(1):60. doi: 10.1007/s10565-024-09903-3 (PMC11286718; doi:10.1007/s10565-024-09903-3)
Supplement: Supplementary file 1 — Supplementary file1 (DOCX 1271 KB) [file 10565_2024_9903_MOESM1_ESM.docx]

**Supporting Information for**

**Original article**

**Proteasome Activity Inhibition Mediates Endoplasmic Reticulum Stress-Apoptosis in Triptolide/Lipopolysaccharide-induced Hepatotoxicity**

Ruohan Cheng^a^, Yihan Jiang^a^, Yue Zhang^a^, Mohammed Ismail^a^, Luyong Zhang^a,b^*, Zhenzhou Jiang^a,c^*, Qinwei Yu^a^*

a New Drug Screening Center, Jiangsu Center for Pharmacodynamics Research and Evaluation, State Key Laboratory of Natural Medicines, China Pharmaceutical University, Nanjing 210009, China

b Center for Drug Research and Development, Guangdong Pharmaceutical University, Guangzhou 510006, China

c Key Laboratory of Drug Quality Control and Pharmacovigilance, Ministry of Education, China Pharmaceutical University, Nanjing 210009, China

*Corresponding authors.

*Email: [lyzhang@cpu.edu.cn](mailto:lyzhang@cpu.edu.cn) (Luyong Zhang);

*Email: [beaglejiang@cpu.edu.cn](mailto:beaglejiang@cpu.edu.cn) (Zhenzhou Jiang); Tel: +(86)2583271043; Fax: +(86)2583271142;

*Email: [yuqinwei7213@cpu.edu.cn](mailto:yuqinwei7213@cpu.edu.cn) (Qinwei Yu)

**1. Material and methods**

*1.1.* **RNA extraction and qPCR**

Total mRNA was extracted from mouse liver using Trizol reagent. The 1 μg/μL RNA was reversed to cDNA after quantifying the RNA concentration with Nanodrop 2000 (Thermo Fisher Scientific, USA). Using AceQ®SYBR Green Master Mix (High ROX Premixed) and Applied Biosystems StepOne™ real-time quantitative PCR (Bio-Rad Laboratories, Hercules, CA, USA). The primers sequences are listed in Table 1. GAPDH was used to standardise the amount of cDNA.

**Table 1** The primer sequences used for qPCR assay in mice

| Gene | Forward primer (5'-3') | Reverse primer (5’-3’) |
| --- | --- | --- |
| *Gapdh* | CATCACTGCCACCCAGAAGACTG | ATGCCAGTGAGCTTCCCGTTCAG |
| *Grp78* | TGTCTTCTCAGCATCAAGCAAGG | CCAACACTTCCTGGACAGGCTT |
| *Atf4* | AACCTCATGGGTTCTCCAGCGA | CTCCAACATCCAATCTGTCCCG |
| *Chop* | GGAGGTCCTGTCCTCAGATGAA | GCTCCTCTGTCAGCCAAGCTAG |

**2. Supporting figures**

**
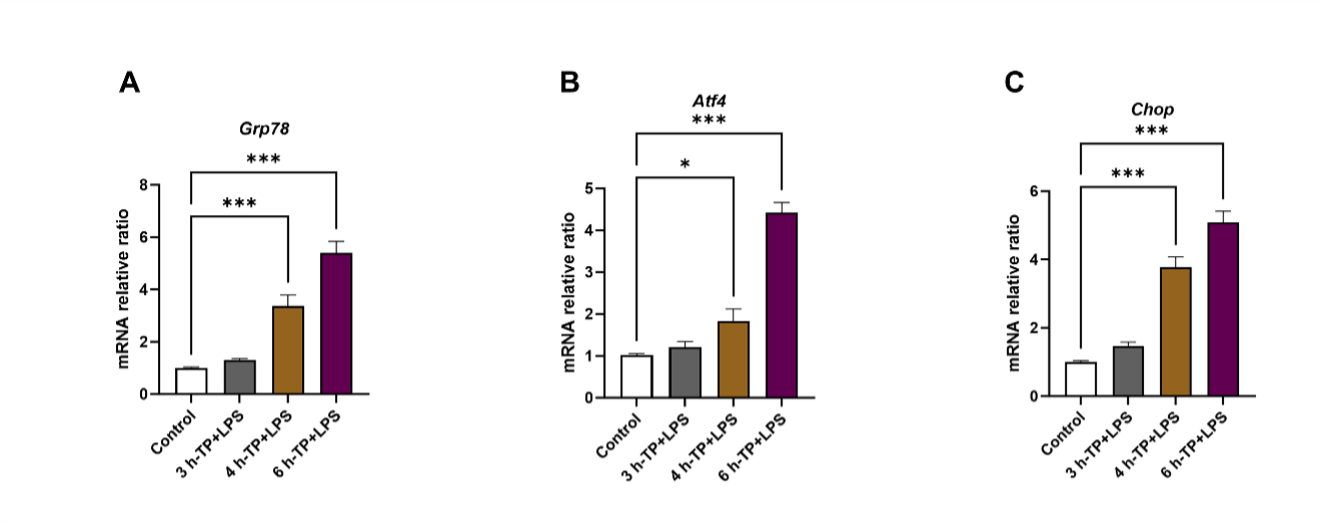
**

**Fig S1 Gene expressions of ERS-related apoptosis.** (A-C) Relative mRNA levels of ERS-related genes, including grp78, atf4, and chop, were detected by qPCR with gapdh as the internal control results (n = 6). Results were expressed as mean ± SEM and statistical analysis was performed using One-Way and Tukey’s multiple comparison test. ^*^P<0.05, ^***^P<0.001, compared with Control group.


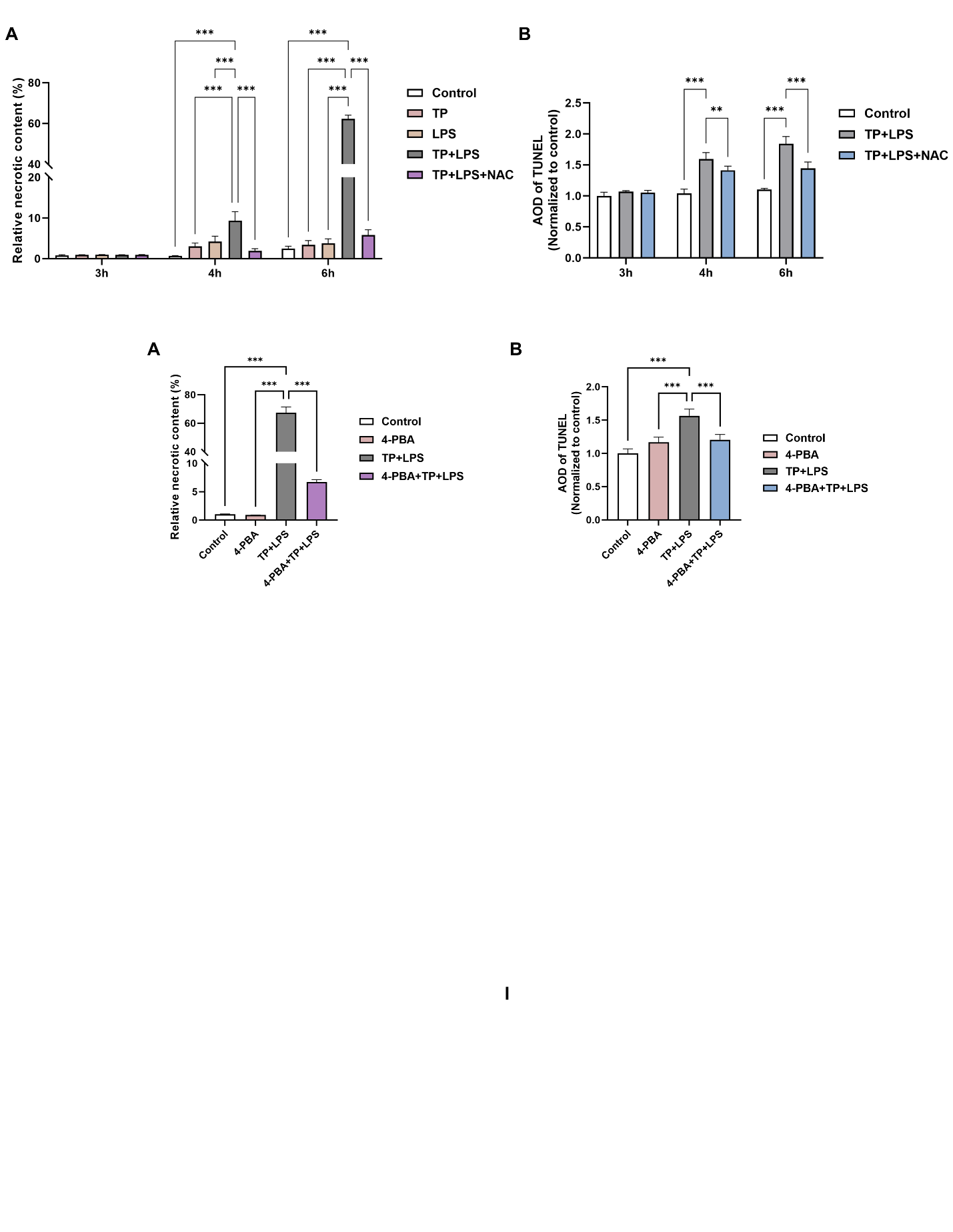


**Fig. S2 Quantifying the change of HE and TUNEL staining at 3, 4, and 6h.** (A) Quantification of HE staining (n=6). (B) Quantification of TUNEL staining (n=6). Average Optical Density is the ratio of Integrated option density and Area(n=6). Results were normalized to control and statistical analysis was performed using One-way ANOVA following by Tukey’s multiple comparison test. ^**^P<0.01, ^***^P<0.001, compared with TP+LPS group.

**
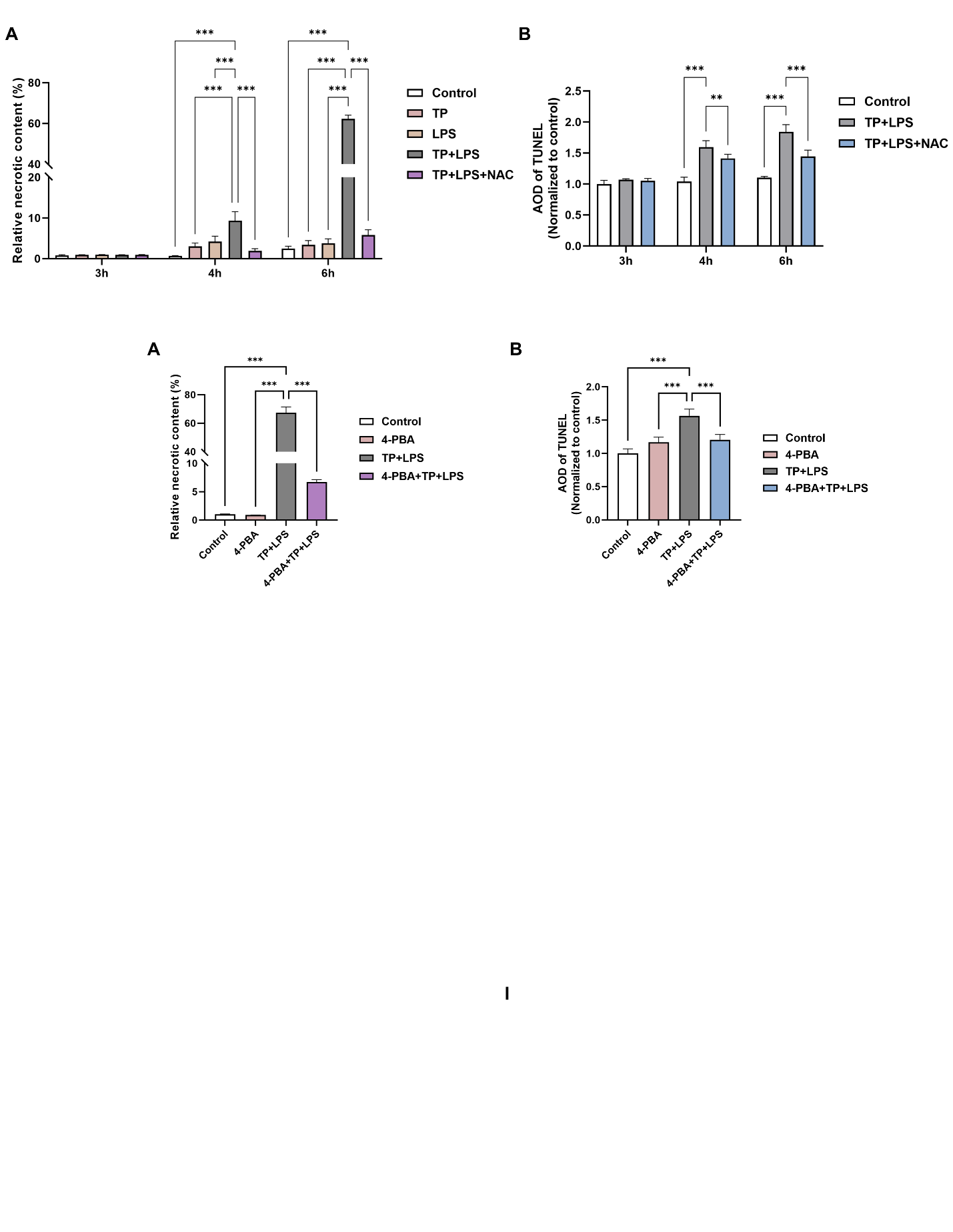
**

**Fig. S3** **Quantifying the change of HE and TUNEL staining**. (A) Quantification of HE staining (n=6). (B) Quantification of TUNEL staining (n=6). Average Optical Density is the ratio of Integrated option density and Area (n=6). Results were normalized to control and statistical analysis was performed using One-way ANOVA following by Tukey’s multiple comparison test. ^***^P<0.001, compared with TP+LPS group.

**
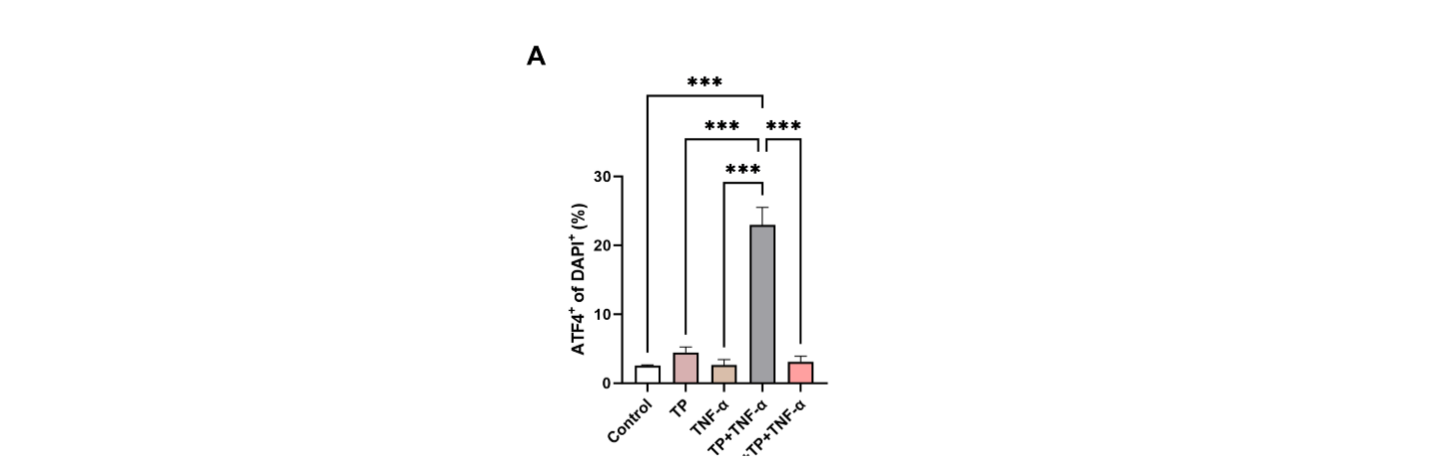
**

**Fig. S4 Quantifying the change of immunofluorescence**. (A) The percentage of DCFH-DA^+^ cells (n=3). Statistical analysis was performed using One-way ANOVA following by Tukey’s multiple comparison test. ^***^P<0.001, compared with TP+TNF-α group.

**
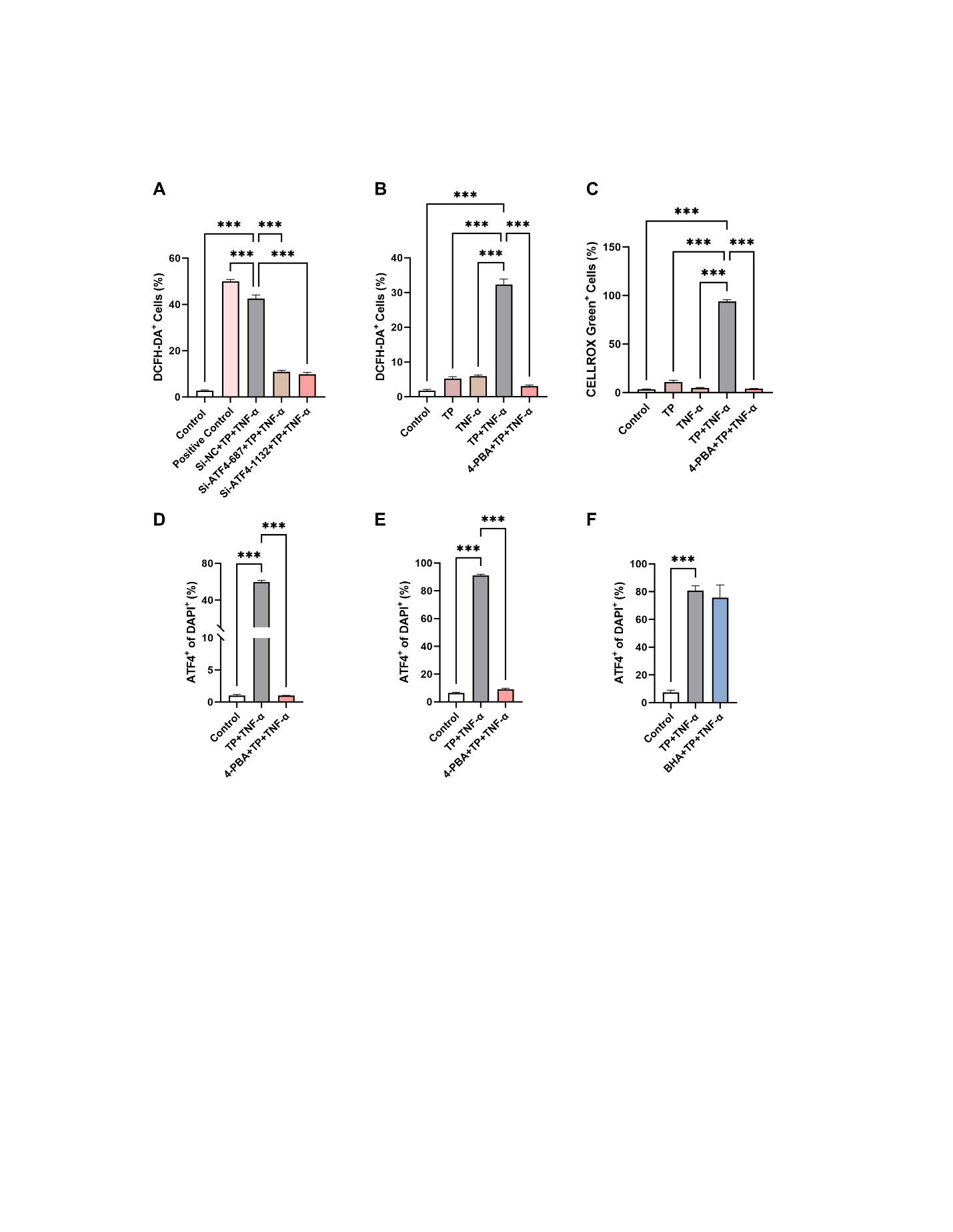
**

**Fig. S5 Quantifying the change of immunofluorescence.** (A-B) The percentage of DCFH-DA^+^ cells (n=3). (C) The percentage of CELLROX Green^+^ cells (n=3). (D-F) The percentage of ATF4^+^ cells (n=3). Statistical analysis was performed using One-way ANOVA following by Tukey’s multiple comparison test. ^***^P<0.001, compared with TP+TNF-α group.

**
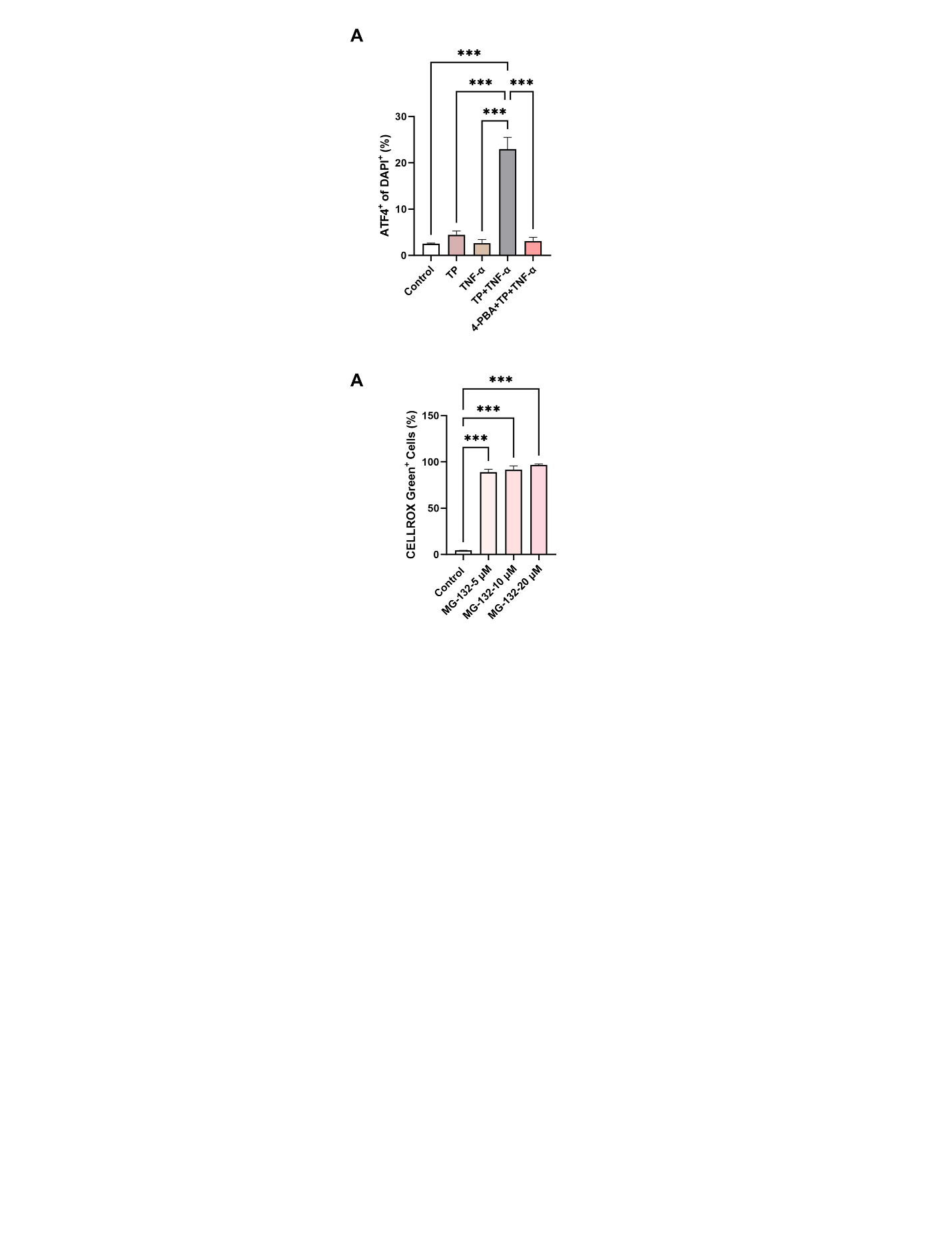
Fig. S6 Quantifying the change of immunofluorescence.** (A) The percentage of CELLROX Green^+^ cells (n=3). Statistical analysis was performed using One-way ANOVA following by Tukey’s multiple comparison test. ^***^P<0.001, compared with control group.
